# Link node: A method to characterize the chain topology of intrinsically disordered proteins

**DOI:** 10.1002/qub2.96

**Published:** 2025-03-16

**Authors:** Danqi Lang, Le Chen, Moxin Zhang, Haoyu Song, Jingyuan Li

**Affiliations:** ^1^ School of Physics Zhejiang University Hangzhou China; ^2^ Department of Physics École Normale Supérieure Paris France

**Keywords:** chain topology, gauss linking number, intrinsically disordered protein, physical link

## Abstract

Intrinsically disordered proteins (IDP) are highly dynamic, and the effective characterization of IDP conformations is still a challenge. Here, we analyze the chain topology of IDPs and focus on the physical link of the IDP chain, that is, the entanglement between two segments along the IDP chain. The Gauss linking number of two segments throughout the IDP chain is systematically calculated to analyze the physical link. The crossing points of physical links are identified and denoted as link nodes. We notice that the residues involved in link nodes tend to have lower root mean square fluctuation (RMSF), that is, the entanglement of the IDP chain may affect its conformation fluctuation. Moreover, the evolution of the physical link is considerably slow with a timescale of hundreds of nanoseconds. The essential conformation evolution may be depicted on the basis of chain topology.

## 1 INTRODUCTION

Intrinsically disordered proteins (IDPs) are involved in various biological functions such as signal transduction, gene regulation, and cell differentiation [1, 2]. IDPs are highly dynamic [3, 4], which are believed to be related to their functionality [5, 6]. Moreover, the conformational space of IDPs is highly complex [7]. Although some indicators such as the radius of gyration (*Rg*) [8, 9] and asphericity (*Asphe*) [10, 11] are commonly used to describe the IDP conformation, they fail to provide a comprehensive depiction of IDP structures. For example, Figure S1 shows two conformations of a typical IDP—the RGG domain of LAF‐1 [12, 13]. Although the values of *Rg* and *Asphe* are comparable between these two conformations (with *Rg* being 2.61 nm and *Asphe* being 0.07 and 0.08 nm, respectively), their structures are apparently different. Specifically, one conformation exhibits compaction in the C‐terminal region (Figure S1A, left panel), whereas the other exhibits compaction in the middle region (Figure S1B, left panel). Accordingly, *Rg* and *Asphe* are not sufficient to fully characterize the structures of IDPs.

As revealed in previous studies, topology analysis is an effective approach to characterize the unconventional structures of proteins [14]. This method aims to characterize the topological structures of peptide chains [15], such as links [16, 17, 18], knots [19, 20, 21, 22, 23], and lassos [24, 25, 26] as well as related pseudotopological structures [27], such as physical links [28] and pseudo knots [29]. The depiction of chain topology is conceived as a supplement of the canonical methods (e.g., secondary structures [30]). It should be noted that the structures of IDPs are irregular with the absence of secondary structures. Therefore, it is interesting to characterize the structures of IDP with regard to their chain topology.

**FIGURE 1:**
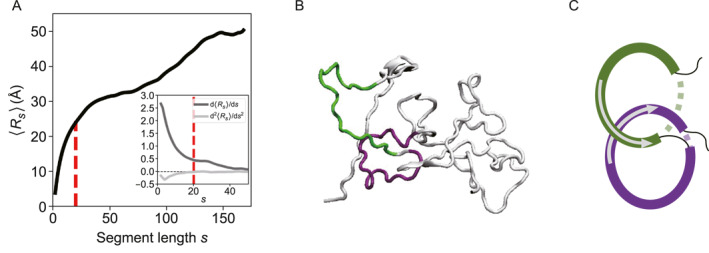
Defining sub‐chains in the LAF‐1 RGG domain and the corresponding physical link. (A) Average inter‐residue distance 〈*R*_*s*_〉 profile. Inset: the first derivative and second derivative of 〈*R*_*s*_〉, where *s* = 20 serve as an inflection point (red dashed line). (B) A representative physical link with the segment length *s* = 20. (C) The corresponding schematic picture.

Here, we proposed to analyze the physical link within the IDP chain, that is, the entanglement between two segments in the IDP chain. More specifically, the segments of the IDP chain are regarded as directed sub‐chains, and their intertwining is estimated by the classical Gauss linking number (*GLN*) method. *GLN*—or mutual writhe value [31]—can depict the relative spatial relationship between two directed sub‐chains and measure how one sub‐chain winds around the other [32]. The *GLNs* of two standard links (formed by two closed circles) are +1 and −1 (according to their relative direction, Figure S2) [33]. Because the sub‐chains of IDP have open tails, they cannot form a standard link; instead, they intertwine with each other (i.e., forming the physical link, Figure 1B,C). The corresponding *GLN* thereby depicts their similarity to the standard link [34]. On the basis of the *GLN* method, the relative spatial relationship of various pairs of sub‐chains throughout the whole IDP chain is then systematically studied [31]. In addition, the resulting *GLN* map can effectively depict the chain topology of IDP and illustrate all physical links. The crucial residues of the physical link, the site where two sub‐chains intersect, are then identified and denoted as the link node. The IDP conformation and its evolution are then characterized with regard to the identified link node.

In this work, we use this method to characterize the conformation of a typical IDP—the LAF‐1 RGG domain. The conformations are obtained from all‐atom molecular simulation. We compute the *GLN* maps of the LAF‐1 RGG, as well as the corresponding link nodes. We noticed that the profile of link node probability is highly heterogeneous and there are certain residues that largely affect the whole chain topology of IDP. We further analyzed the conformation evolution of IDP on the basis of *GLN* maps. Notably, the timescale of the evolution can reach hundreds of nanoseconds; the chain topology of IDP is considerably stable which is distinct from the flipping of the residue contact. In summary, the *GLN* map with link node can serve as an effective method to depict the IDP conformation and its evolution.

## 2 MODELS AND METHODS

### 2.1 Physical link

The chain topology can be characterized by links, which is composed of two closed chains connected to each other (Figure S2). For open chains, pseudo‐topology [27] is then developed to characterize their structures, which utilize the physical link [28, 35] to depict the intra‐chain entanglement. By artificially closing each sub‐chain, the resulting structure is a standard link (Figure 1C). The orientation of the physical link is consistent with that of the corresponding standard link, which is either positive or negative (Figure S2). In this work, we adopt the physical link to characterize the pseudo‐topology of the IDP chain, thus characterizing the IDP conformation.

### 2.2 *GLN* algorithm

*GLN*—or mutual writhe value—is a standard method to depict physical links [36]. It measures the number of windings of two curves, and the sign reflects the orientation. The definition is as follows:

(1) $$\begin{array}{l} GLN\equiv \frac{1}{4\pi }\oint_{\gamma_{1}}\oint_{\gamma_{2}}\frac{{\vec{r}}^{\left(1\right)}-{\vec{r}}^{\left(2\right)}}{{\left|{\vec{r}}^{\left(1\right)}-{\vec{r}}^{\left(2\right)}\right|}^{3}}\cdot \left(d{\vec{r}}^{\left(1\right)}\times d{\vec{r}}^{\left(2\right)}\right) \end{array}$$

where ${\vec{r}}^{\left(1\right)}$ and ${\vec{r}}^{\left(2\right)}$ are the spatial coordinates of two curves. It should be noted that since the physical link is not a standard link, the *GLN* of the physical link is not an integer, which can serve as the potential of linking. *GLN* = 0 indicates the absence of a physical link.

For proteins, curves are the collections of positions of C‐*α* atoms in two sub‐chains with *N*_1_ and *N*_2_ residues, respectively. *GLN* can approximately be measured by the following Equation [37]:

(2) $$\begin{array}{l} GLN\equiv \frac{1}{4\pi }\sum_{i=1}^{N_{1}-1}\sum_{j=1}^{N_{2}-1}\frac{{\vec{R}}_{i}^{\left(1\right)}-{\vec{R}}_{j}^{\left(2\right)}}{{\left|{\vec{R}}_{i}^{\left(1\right)}-{\vec{R}}_{j}^{\left(2\right)}\right|}^{3}}\cdot \left(d{\vec{R}}_{i}^{\left(1\right)}\times d{\vec{R}}_{j}^{\left(2\right)}\right) \end{array}$$

where we use the midpoint approximation $R_{i}^{\left(k\right)}=\left(r_{i+1}^{\left(k\right)}+{r_{i}}^{\left(k\right)}\right)/2$, and ${r_{i}}^{\left(k\right)}$ represents the coordinates of the *C*_*α*_ atoms in residues i of sub‐chain k.

### 2.3 Defining sub‐chains

We utilize the *GLN* algorithm to depict the physical link, whereas the calculation of *GLN* between sub‐chains within IDP requires a definition of two sub‐chains. To define the length of sub‐chains, we calculate the average interresidue distance $\langle R_{s}\rangle$ with varying segment lengths of s [residues] [38]. Figure 1 presents the $\langle R_{s}\rangle$ profile of a representative LAF‐1 RGG conformation. Both the first derivative and second derivative of the $\langle R_{s}\rangle$ are calculated as shown in the inset of Figure 1A. Clearly, the first derivative reaches a plateau at approximately s = 20, and the second derivative reaches around zero here, suggesting that s = 20 is an inflection point. Figure 1B represents a snapshot of two sub‐chains (with *s* = 20). They are in arc shapes and intend to cross each other, and their *GLN* is about 0.5. In the following, we consider this entanglement as a typical physical link—by artificially closing each sub‐chain, it is a standard Hopf link (Figure 1C).

### 2.4 *GLN* map

We use *GLN*_*i*_,_*j*_ to represent the *GLN* between sub‐chain i (with residue index i−i+s) and sub‐chain j (with residue index j−j+s). There are 168 residues in the LAF‐1 RGG domain. The *GLNs* between various sub‐chains are studied systematically using the Python package Topoly (version 0.9.17) [39]. The resulting 127 × 127 matrix is defined as the *GLN* map, which is denoted as follows:

(3) $$M\equiv \left(\begin{array}{cccc} GLN_{1,22} & GLN_{1,23} & \cdots & GLN_{1,148}\\ & GLN_{2,23} & \cdots & GLN_{2,148}\\ & & \ddots & \vdots \\ & & & GLN_{127,148} \end{array}\right)$$

### 2.5 Preparing the structures of LAF‐1 RGG protein

The procedure of the simulation of LAF is described in our previous study [40]. In order to mimic physiological conditions, sodium and chloride ions are added to neutralize the system, resulting in a 150‐mM NaCl concentration. Five independent 1000‐ns simulations are conducted to characterize the structure of IDPs.

## 3 RESULTS AND DISCUSSION

### 3.1 The *GLN* map of the LAF‐1 RGG domain

**FIGURE 2:**
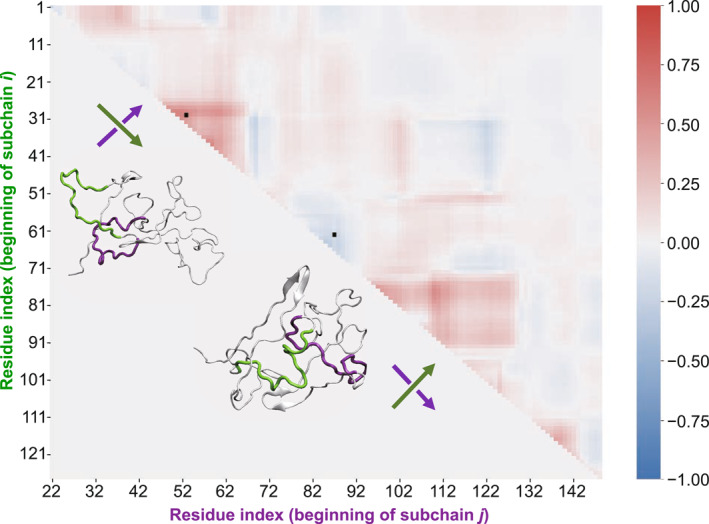
The *GLN* map of a representative conformation. The *x* axis represents the beginning residue of sub‐chain *i*, and the *y* axis represents the beginning residue of sub‐chain *j*. Each cell on the *GLN* map represents the *GLN* of sub‐chain *i* and sub‐chain *j*. Red and blue colors indicate the positive and negative directions, respectively. The corresponding snapshots of the marked cells are represented nearby, where sub‐chain *i* is highlighted in green and sub‐chain *j* is in purple. GLN, Gauss linking number.

We introduce the *GLN* map method to characterize the chain topology of the LAF‐1 RGG domain. Sub‐chains containing 20 residues are considered, and the *GLN* of various pairs of these sub‐chains (denoted by sub‐chain *i* and sub‐chain *j*, respectively) is calculated (see the Models and Methods section for details). One representative *GLN* map and the corresponding conformation are shown in Figure 2. It can be seen that there are several patches in the *GLN* map, especially near the diagonal (marked by black squares), which correspond to the physical links within the IDP chain (see the snapshots in Figure 2). As shown in this representative *GLN* map, there are three apparent patches. This pattern illustrates that there are multiple crossings of sub‐chains and the presence of multiple physical links in IDP conformation. Thus, the chain topology of IDP can be effectively depicted in terms of the arrangement of crossings (i.e., physical links).

It should be noted that the *GLN* map can effectively discriminate the IDP conformations with similar *Rg* and *Asphe*. Figures S1A,B show the *GLN* maps of the two IDP conformations with comparable *Rg* (about 2.61 nm) and *Asphe* (about 0.07). The patterns of these two *GLN* maps are apparently different. As for the conformation in Figure S1A, the corresponding *GLN* map shows a typical patch in its upper left corner (corresponding to the N‐terminal part of the RGG domain), indicating the presence of a physical link in this region. On the other hand, there is no patch in this region of Figure S1B. Hence, the chain topology of these two IDPs is different, even though they share similar profiles.

Moreover, the evolution of IDP conformation can be analyzed with regard to the change in chain topology on the basis of the trajectory of *GLN* maps. Figure S4 shows *GLN* maps at *t* = 600 ns, 750 ns, and 900 ns of a representative trajectory. At *t* = 600 ns, there is a physical link between sub‐chains 61–81 and 87–107; at *t* = 750 ns, these two sub‐chains become parallel and the physical link is released; and at *t* = 900 ns, they form a physical link again but with the reverse orientation. In other words, their spatial relationship is reversed. The conformation change can be attributed to the rearrangement of the chain topology of IDP. Hence, the analysis of chain topology may provide useful insights into the conformation evolution of IDP.

### 3.2 Identification of the link node in the RGG domain

In the *GLN* map, there is emergence of color patches, that is, the regions with a considerable color gradient. This alternation of chain topology can be quantitatively reflected in the *GLN* changing from zero to nonzero. Specifically, the change of the amplitude of *GLN* is calculated and denoted as ∆|*GLN*|

(4) $$\Delta |GLN_{i,j}^{h}|\;=\;\frac{|GLN_{i,j+1}\left|-\right|GLN_{i,j-1}|}{|GLN_{i,j}|}$$

(5) $$\Delta |GLN_{i,j}^{v}|\;=\;\frac{|GLN_{i+1,j}\left|-\right|GLN_{i-1,j}|}{|GLN_{i,j}|}$$

where ∆|*GLN*|^*h*^ and ∆|*GLN*|^*v*^ represent the change of |*GLN*|^*h*^ in the horizontal and vertical axis of the *GLN* map, respectively. ∆|*GLN*| is then exploited to identify the boundary of a patch. Firstly, the sites with large ∆|*GLN*| are identified according to the criterion ∆|*GLN*| > 50% (see Supporting Information Section 4 for details). Secondly, those consecutive sites are chosen, and the corresponding line of these sites is then identified as the boundary of a patch. Boundaries with a length beyond 20 (the plateau of the 〈*R*_*s*_〉 profile is around 20, Figure 1A) are considered in the following discussion. Let us take the red patch in Figure 2 (marked by a black square) as an example. The sites with |*GLN*|^*v*^ exceeding 50% are identified (see the enlarged *GLN* map in Figure S7). These sites are consecutive and located at line *i* = 27 (Figure S7). Thus *i* = 27 can be considered as a horizontal boundary of this color patch, and the length of this boundary line is beyond 20. Similarly, the identified vertical boundary is *j* = 67 (Figure S8).

The residues in these boundaries are further discussed on the basis of the sign of ∆|*GLN*|. If ∆|*GLN*| > 0, the terminal residue of the corresponding sub‐chain (i.e., closest to the C‐terminus) is about to cross with another sub‐chain. For instance, line *i* = 27 indicates that the terminal residue of sub‐chain 27–47 is about to cross with the other sub‐chain (Figure S8A). Likewise, if ∆|*GLN*| < 0, it indicates that the beginning residue of this sub‐chain (i.e., closest to the N‐terminus) is about to cross with the other sub‐chain. For instance, line *j* = 67 indicates that the beginning residue of sub‐chain 67–87 is about to cross with the other sub‐chain (Figure S8C). Therefore, these residues serve as crossing points [41] of the physical link and are then denoted as link nodes (see the algorithm of link node identification in SI, Figure S6).

In summary, on the basis of the *GLN* map, we analyze the boundary of the patch and identify the corresponding link node (e.g., residues 47 and 67). These link nodes are located at the sites where two sub‐chains cross and effectively depict the corresponding physical link. Hence, link nodes can be regarded as pivotal residues to depict the chain topology of IDP.

### 3.3 Characterization of IDP structures on the basis of link nodes

**FIGURE 3:**
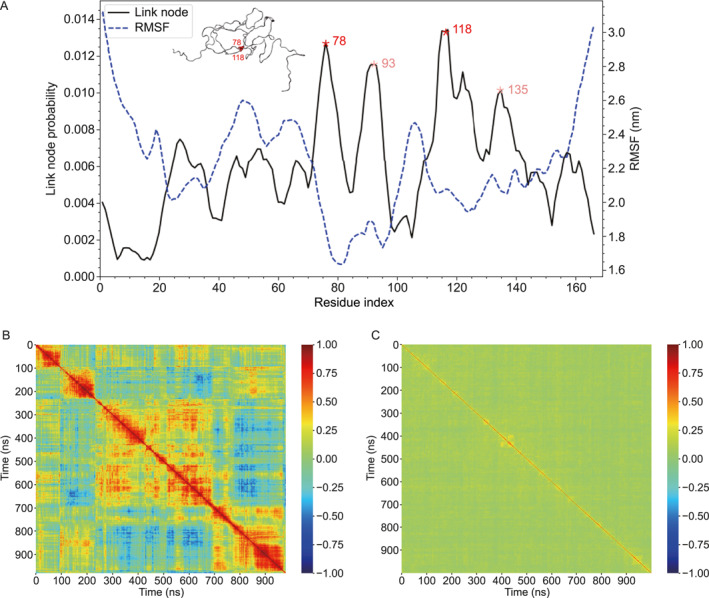
The link node probability distribution and the evolution of *GLN* map. (A) The link node probability distribution (black line) and the RMSF (blue line). The snapshot of a representative conformation reveals that 78 and 118 are the crossing points. (B) The correlation coefficient matrix of *GLN* maps. (C) The correlation coefficient matrix of contact maps. *GLN*, Gauss Linking Number.

Link nodes are then exploited to analyze the conformation evolution of the LAF‐1 RGG domain, and five independent 1000‐ns trajectories are considered. The link node probabilities of these five trajectories, that is, the probability distributions of residues involved in link nodes, are calculated. Figure 3A shows the probability profile of one trajectory (the other four probability profiles are represented in Figure S13). These probability profiles are highly heterogeneous. As shown in Figure 3A, the propensity of some residues, that is, 78Asp (1.20%), 93Arg (1.15%), 118Gly (1.34%), and 135Asn (1.01%), is about twice the average (0.58%). A representative conformation illustrates the case that 78Asp and 118Gly serve as link nodes (Figure 3A, upper left corner).

To further illustrate the contribution of link node residues to the conformation evolution, the RMSF of each residue is calculated and compared with the link node probability. The RMSF and link node probability exhibit an anticorrelation with a Pearson correlation coefficient of −0.55. Such a relationship is also observed in the other four trajectories with correlation coefficients of −0.56, −0.47, −0.40, and −0.51, respectively (Figure S13). Notably, the regions with high link node propensity (e.g., 78Asp, 93Arg, 118Gly, and 135Asn) usually have low RMSF (Figure 3A). The RMSFs of these residues are less than 2.0 nm, which is well below the average value (2.2 nm). In other words, the regions involved in link nodes tend to have suppressed structural fluctuation. Therefore, the link node method should also provide useful information about the topological constraint imposed on the residues during the conformation fluctuation of IDP.

The conformation fluctuation of IDP is further analyzed from the perspective of chain topology. The evolution of chain topology is analyzed by calculating the correlation coefficient (*ρ*) among *GLN* maps. *ρ* is defined as the inner product of two *GLN* maps and normalized by the Frobenius norm:

(6) $$\rho_{M\left(t_{1}\right),\;M\left(t_{2}\right)}=\frac{\langle M\left(t_{1}\right),M\left(t_{2}\right)\rangle }{∥M\left(t_{1}\right){∥}_{F}\cdot ∥M\left(t_{2}\right){∥}_{F}}$$

where *M* (*t*) is the *GLN* map at *t*. The resulting correlation matrix is shown in Figure 3B. The correlation coefficient between the *GLN* map with close instant is considerably high. Several blocks (with *ρ* maintaining up to 0.75) are identified along the diagonal of the correlation matrix. Moreover, the timescale of these blocks generally reaches hundreds of nanoseconds. This suggests that the evolution of the chain topology of IDP is quite slow. There are sustained physical links that are involved in the conformation evolution of IDP. Figure S11 shows the *GLN* maps at *t* = 310, 350, and 390 ns, which are in the same block in Figure 3B. Clearly, the physical links 70–90 and 110–130 are sustained during conformation evolution. This indicates that although the IDP undergoes significant conformational fluctuations, it still maintains a similar chain topology. Additionally, the correlation matrices of the *GLN* map of the other four trajectories are shown in Figure S14 and the timescale of the diagonal blocks can also reach hundreds of nanoseconds. A relatively slow evolution of the chain topology of IDP is also observed in these trajectories.

The probability distribution of residue contacts is also calculated for comparison. The probability profile is rather homogeneous (Figure S9). There are far more residue contacts in IDP structures: about 50 residue contacts in each conformation, whereas there are only about 5 pairs of link nodes (Figure S10). In other words, the homogeneity of abundant residue contacts makes it less effective to depict IDP structures. Besides, we also calculate the correlation coefficients between contact matrices to reveal the evolution of residue contacts (Figure 3C). Clearly, their correlation coefficients are generally low, and there are no blocks that can be identified on the correlation matrix. This suggests that the evolution of the residue contact is fast and it cannot characterize the sustained feature during the conformation evolution of IDP.

Interestingly, in the study of the chain topology of structured proteins, previous literature reveals that the folding rate of proteins is negatively correlated with entanglement between sub‐chains [42]. This aligns with our finding that the link node is negatively correlated with structure fluctuations. The entanglement in their work is depicted by the maximum *GLN* of sub‐chains, which is consistent with the concept of the link node.

### 3.4 Mechanism underlying sustained chain topology

**FIGURE 4:**
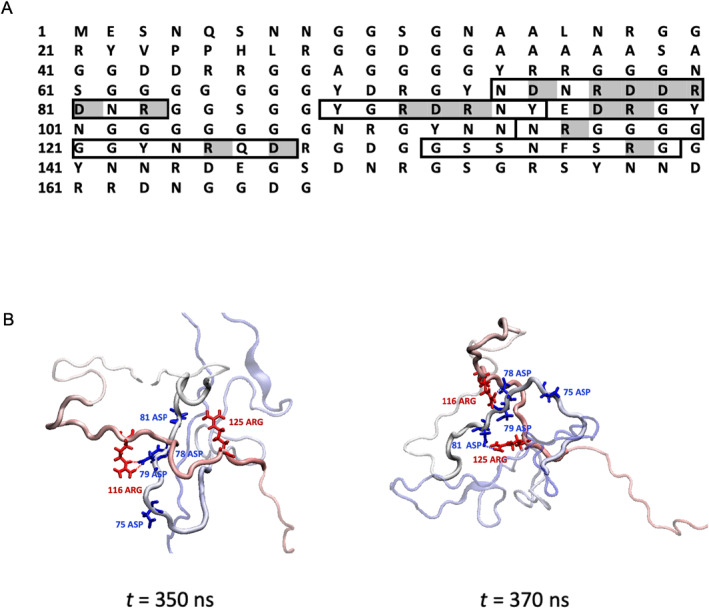
The interaction mode for the sustained physical link. (A) The sequence of the LAF‐1 RGG domain. Four segments with higher link node propensity (74–83, 89–95, 115–127, and 132–139) are outlined by rectangles. Charged residues are highlighted in gray. (B) Snapshots of representative sustained physical link. The IDP is colored in blue to red (from N‐terminus to C‐terminus), and segments 74–83 and 115–127 are highlighted in bold. IDP, Intrinsically disordered proteins.

As revealed by our results about the correlation coefficient matrix of the *GLN* map, the evolution of the chain topology of IDP is rather slow. To study the mechanism underlying such sustained chain topology, the interaction modes associated with physical links are then discussed. In the representative trajectory, the sustained physical links with the lifetime >50 ns are identified and the probability distribution of the link nodes of sustained physical links is shown in Figure S12. Interestingly, the distribution of the sustained link node is similar to the overall link nodes. The residues with higher link node propensity are further discussed, that is, larger than 1.0%. There are 38 residues that can be categorized into four segments, that is, 74–83, 89–95, 115–127, and 132–139, which are highlighted in gray in Figure 4A. We notice that there are a total of 14 charged residues (eight Arg and six Asp). The corresponding proportion of charged residues (37%) is considerably higher than the overall proportion of the protein (26%). Figure 4B shows a representative sustained physical link involving segments 74–83 and 115–127. There are four negatively charged residues (75Asp, 78Asp, 79Asp, and 81Asp) in segment 74–83 and two positively charged residues (116Arg and 125Arg) in segment 115–127. The interaction between two segments and the sustained physical link is the electrostatic attraction between these residues. At *t* = 350 ns, the physical link is attributed to the interaction of 79Asp–116Arg; at *t* = 370 ns, the physical link is attributed to both 78Asp–116Arg and 81Asp–125Arg. Overall, the electrostatic attraction mediates the formation and maintenance of such sustained physical links, even though the given interactions of charged residues are highly dynamic.

## 4 CONCLUSION

In this work, we characterize the conformation of IDP based on its chain topology. The *GLN* between the sub‐chains is calculated throughout the IDP chain. The resulting *GLN* maps can depict the corresponding physical links within the IDP chain, thus effectively characterizing its chain topology. The crossing point of the physical link is denoted as the link node. Link nodes can serve as the key residue to depict the chain topology of IDP. We thus employ this link node method to analyze the MD simulation trajectories of the LAF‐1 RGG domain. The formation of link nodes and the interaction between the corresponding sub‐chains can be attributed to the electrostatic interaction between these charged residues. The link node propensity is negatively correlated with RMSF: the structural fluctuation of the link node is suppressed under the topological constraint. Moreover, the conformational evolution of IDP can be depicted on the basis of the evolution of *GLN* map. Notably, the *GLN* Map can remain considerably correlated over hundreds of nanoseconds. Accordingly, the chain topology exhibits considerable dynamic sustainability. Taken together, the link node based on the *GLN* map can serve as an effective method to characterize the IDP conformation and its evolution.

## AUTHOR CONTRIBUTIONS

**Danqi Lang**: Formal analysis; methodology; writing– original draft. **Le Chen**: Writing–review and editing. **Moxin Zhang**: Investigation. **Haoyu Song**: Investigation. **Jingyuan Li**: Project administration; supervision; funding acquisition; writing–review and editing.

## CONFLICT OF INTEREST STATEMENT

The authors Danqi Lang, Le Chen, Moxin Zhang, Haoyu Song, and Jingyuan Li declare that they have no conflicts of interest.

## ETHICS STATEMENT

This article does not contain any studies with human or animal subjects performed by any of the authors.

## Supporting information

Supporting Information S1

## Data Availability

The data that support the findings of this study are available from the corresponding author upon reasonable request.
