# Supplementary material for: Link node: A method to characterize the chain topology of intrinsically disordered proteins
Source: Quant Biol. 2025 Mar 16;13(3):e96. doi: 10.1002/qub2.96 (PMC12806083; doi:10.1002/qub2.96)
Supplement: Supplementary file 1 — Supporting Information S1 [file QUB2-13-e96-s001.pdf]

ORIGINAL ARTICLE

Supporting Information

Danqi Lang[1,2] | Le Chen[1] | Moxin Zhang[1] | Haoyu Song[1] | Jingyuan Li[1*]

Conformations of similar *Rg* and *Asphe*

Figure S1 shows two representative conformations with similar *Rg* and *Asphe*, while their GLN Maps are different.

Gauss Linking Number (GLN)

Figure S2 shows a typical link – Hopf. Link and the corresponding Gauss Linking Number (GLN).

The color intensity of the GLN Map

Figure S3 is an inset of Figure 2 in the main text. It can be seen that from site a to c (i.e., i varies from 26 to 30), sub-chain *i* (green, right panel) pokes deeper into the fixed sub-chain *j*(purple, right panel, with j = 51). This is revealed by the increase of the color intensity (corresponding GLN increases from 0.189 to 0.496).

Revealing the dynamic process by GLN Map

See Figure S4.

The probability distribution of GLN and ΔGLN

Figure S5A-C shows the GLN Map, the absolute vertical gradient (i.e., $GLN_{i+1,j} - GLN_{i-1,j}$) of this GLN Map, and the relative vertical gradient (i.e., $(GLN_{i+1,j} - GLN_{i-1,j})/GLN_{i,j}$), respectively. Both absolute gradient and relative gradient can depict the alternation of the original GLN Map. Here we choose the relative gradient to depict the boundary of patch throughout the manuscript. The probability distribution of the relative gradient of GLN is further calculated (Figure S5F) (for comparison, we also represent the probability distribution of the absolute gradient in Figure S5E. It can be well fitted by the double-gaussian function $a_1 \exp(-\frac{x^2}{2\sigma_1^2}) + a_2 \exp(-\frac{x^2}{2\sigma_2^2})$ (Figure S5F, green line) with the fitting parameter $\sigma_1 = 0.041, \sigma_2 = 0.453$. So we set a criterion $\Delta GLN > 0.5$ to pick out residues falling into the heavy tail of the distribution, thus to depict the boundary of patch.

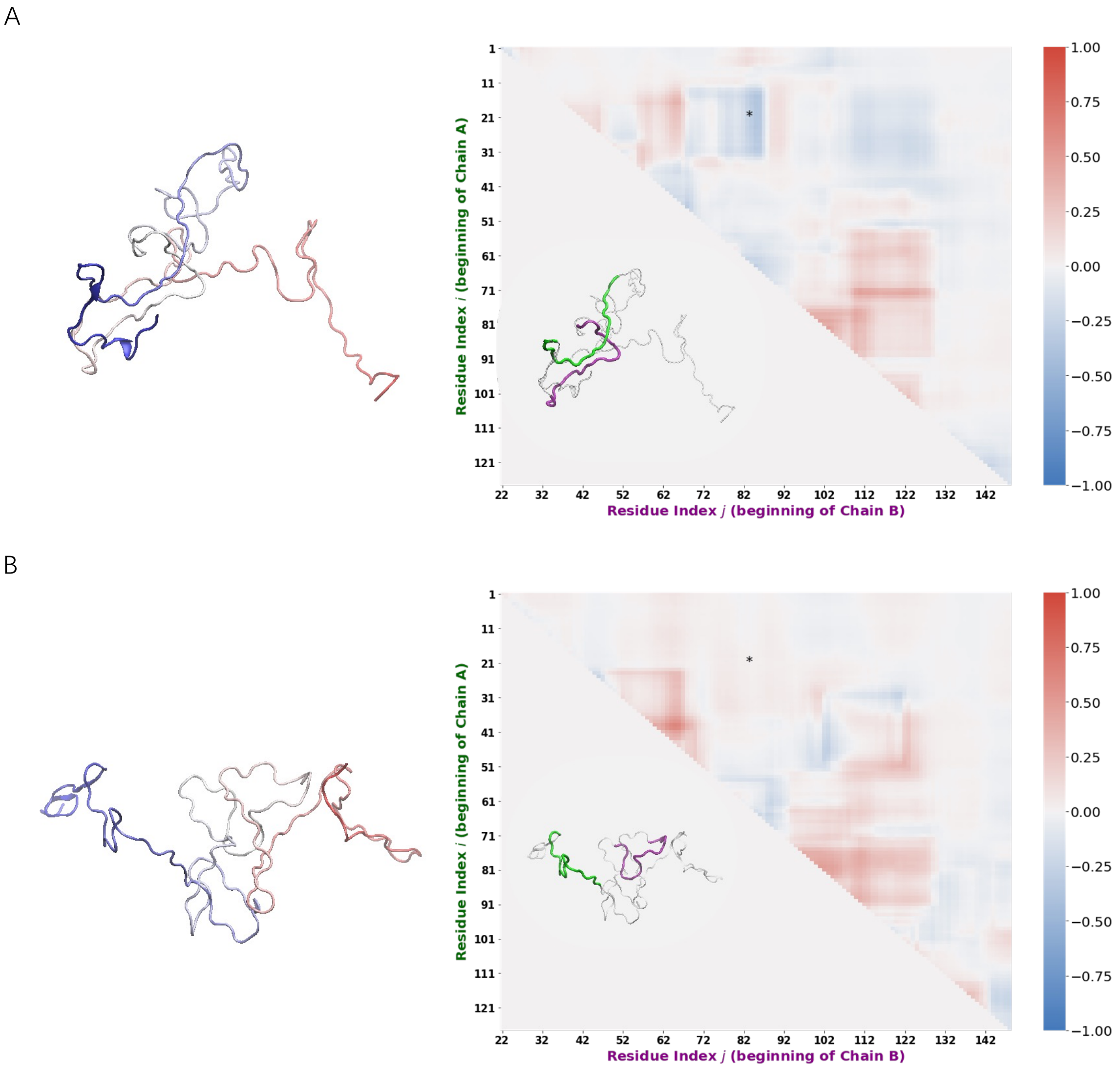

FIGURE S1 Conformations of similar *Rg* and *Asphe* with the corresponding GLN Maps. (A) *Rg* = 2.61nm, *Asphe* = 0.07; (B) *Rg*= 2.61nm, *Asphe* = 0.08.

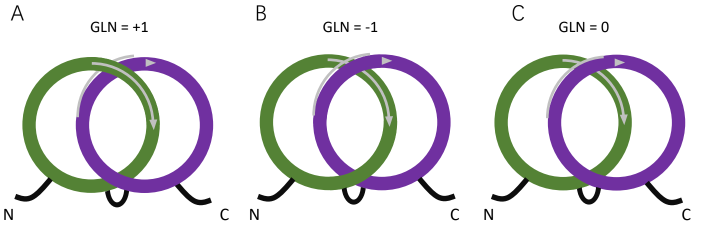

FIGURE S2 Gauss Linking Number (*GLN*). (A) Hopf.1 Link, *GLN*= +1, positive direction. (B) Hopf.2 Link,*GLN* = -1, negative direction. (C) No link,*GLN* = 0.

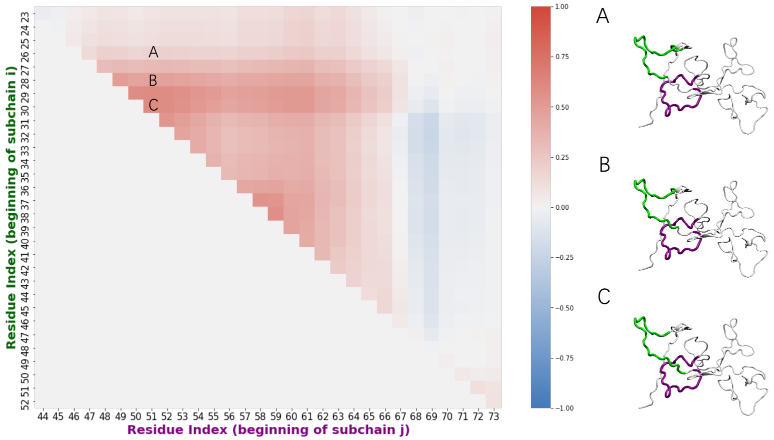

FIGURE S3 Interpretation of the color intensity of *GLN* Map. Left Panel: an inset of the *GLN* Map shown in Figure 2. Right panel: Corresponding Snapshots. sub-chain *i* is in green, sub-chain *j* is in purple. (A) *GLN* = 0.189. (B) *GLN* = 0.327. (C) *GLN* = 0.496.

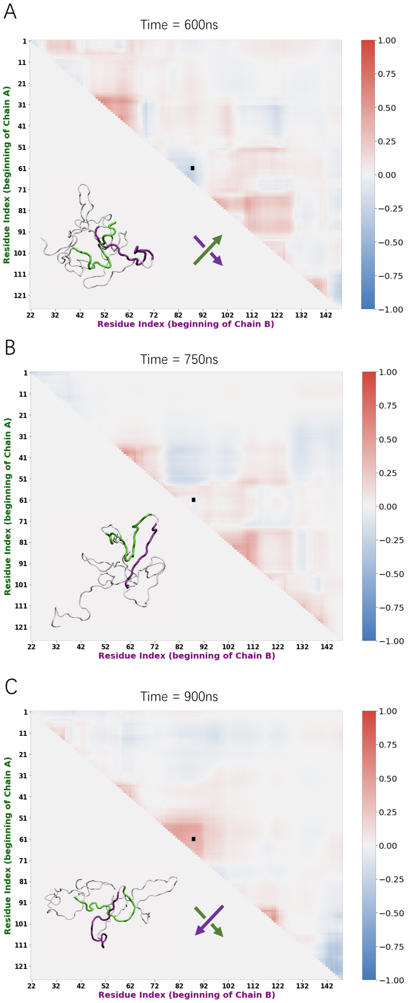

FIGURE S4 *GLN* Map reveals the dynamic process of a typical physical link. (A) t = 600 ns, the physical link is formed in the negative direction. (B) t = 750 ns, the physical link is released. (C) t = 900 ns, the physical link is formed in the positive direction.

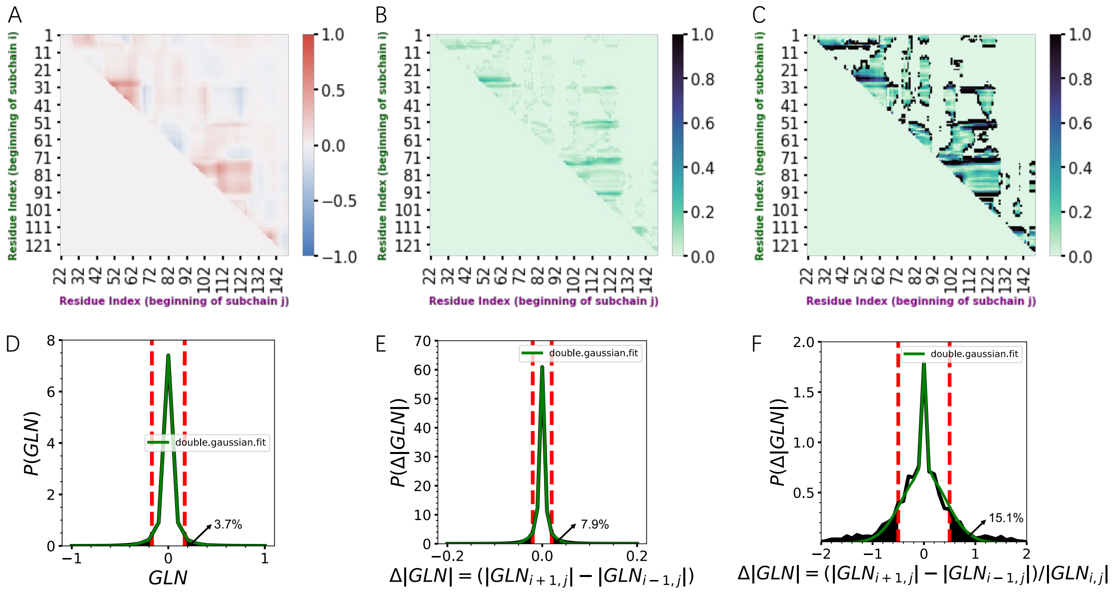

FIGURE S5 (A) Figure **??** in the main text. Each cell corresponds to $GLN_{i,j}$. (B) The plot of absolute gradient $GLN_{i+1,j} - GLN_{i-1,j}$. (C) The plot of relative gradient$(GLN_{i+1,j} - GLN_{i-1,j})/GLN_{i,j}$. (D) The probability distribution of $GLN_{i,j}$ of all conformations. (E) The probability distribution of absolute gradient $GLN_{i+1,j} - GLN_{i-1,j}$. (F) The probability distribution of relative gradient$(GLN_{i+1,j} - GLN_{i-1,j})/GLN_{i,j}$

.

Identification of the Link Node

The process chart for Link Node identification is presented in Figure S6. Firstly, we calculate $\Delta|GLN|$ to represent the variation of $|GLN|$. Then we identify sites with $\Delta|GLN|$ exceeding 50% and the corresponding line is regarded as the boundary. We focus on the boundary with a length over 20 and Link Node is identified on the basis of the sign of ΔGLN of the corresponding boundary (Figure S7).

Accordingly, the boundaries of the red patch of Figure 2 are shown in Figure S8. The line corresponding to the horizontal boundary (i = 27) and vertical boundary (j = 67) cross at site c, thus this patch can be delineated by the dashed gray line $\overline{ac}$ and $\overline{bc}$. For $\overline{ac}$, the corresponding $|GLN| > 0$, indicates the presence of a physical link between sub-chain i and sub-chain j in this region. Within a modestly long variation of sub-chain j (i.e. j ranges from 48 to 68), the terminal residue of sub-chain i, Residue 47 (i.e., i = 27, plus the sequence length 20, the terminal residue is 47) is crossing through sub-chain j (Figure S8A and Figure S8C). As sub-chain i moves towards the C-terminus, the entanglement between sub-chain i and sub-chain j becomes deeper, which is reflected as $\Delta|GLN| > 0$ on $\overline{ac}$. On the other hand, for bc, the $|GLN|$> 0, Figure S8B and S8C display that the beginning residue of sub-chain j, Residue 67, crosses through sub-chain i and thus forms the physical link. If sub-chain j continues to move towards the C-terminus, sub-chain i and sub-chain j will no longer cross, and physical links cannot exist anymore, which is reflected as ΔGLN <0 on $\overline{bc}$. Thus, these two crossing residues (47 and 67) are denoted as Link Nodes. Taken together, Link Node can be identified on the basis of the sub-chain corresponding to the boundary. If the ΔGLN of the boundary line is positive (reflected as color appears), Link Node is the terminal residue of the corresponding sub-chain; if the $\Delta|GLN|$ of the boundary line is negative (reflected as color disappears), Link Node is the beginning residue of the corresponding sub-chain.

Comparison of Link Node and contact method

Figure S9 compares the probability profile of Link Nodes and contacts. Figure S10 compares the evolution of Link Node and contact events.

Sustained physical link

Figure S11 shows a representative sustained physical link.

Other trajectories

Figure S14 shows the probability profile of Link Nodes in the other four trajectories.

Interaction Modes

Figure S15 shows an interaction mode between ARG and ASP.

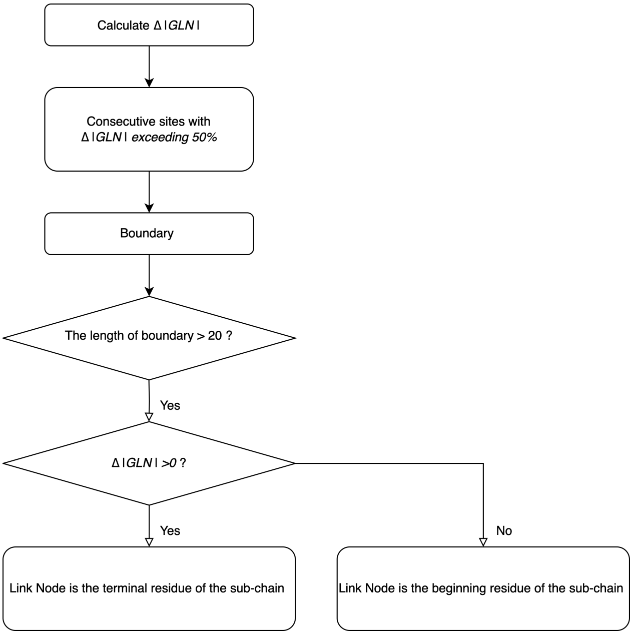

FIGURE S6 The process of Link Node identification.

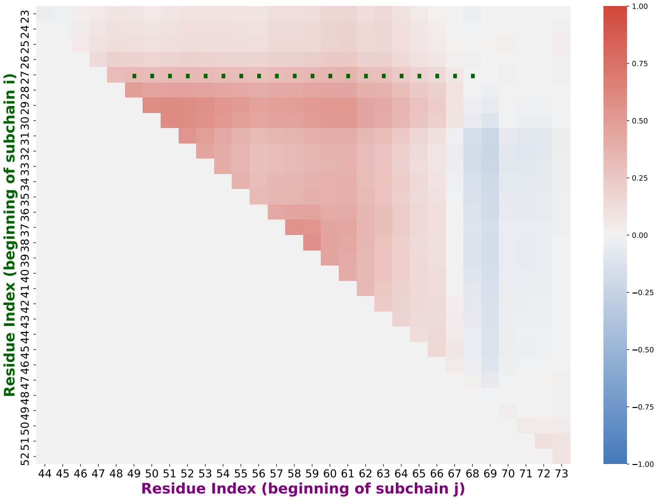

FIGURE S7 Enlarged Figure of Figure 2. Square: Sites with $\Delta|GLN|$ exceed 50%.

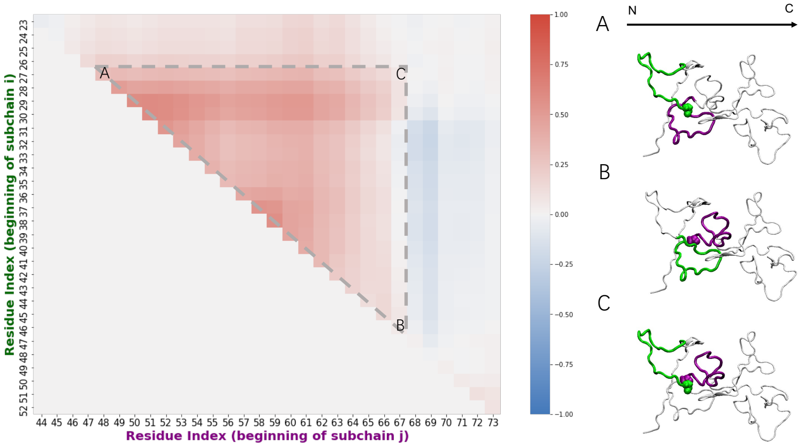

FIGURE S8 The boundary of a representative patch. Residue 47 (green) and residue 67 (purple) are shown in VDW spheres. (A)The terminal residue of sub-chain *i* (47) is the crossing point. (B) The beginning residue of sub-chain *j* (67) is the crossing point. (C) The crossing pair.

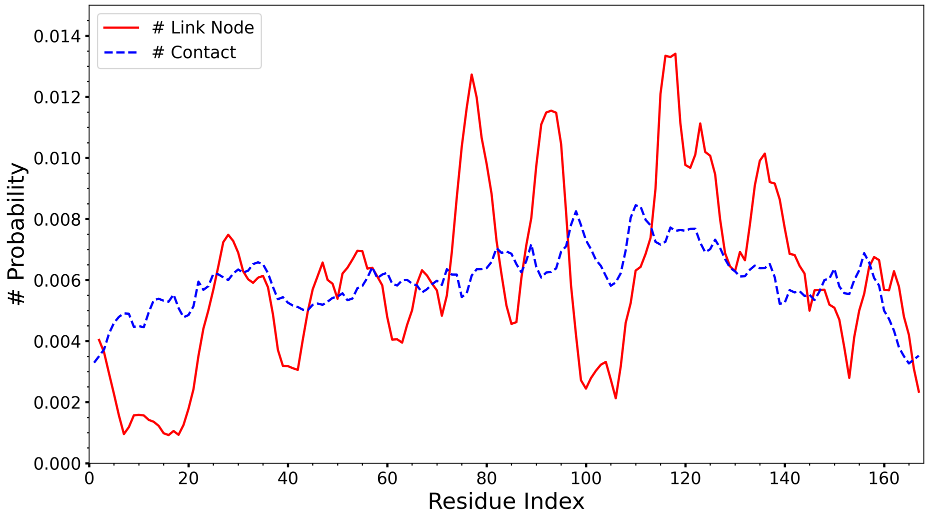

FIGURE S9 The Link Node (red) and contact (blue) probability of sequence profile.

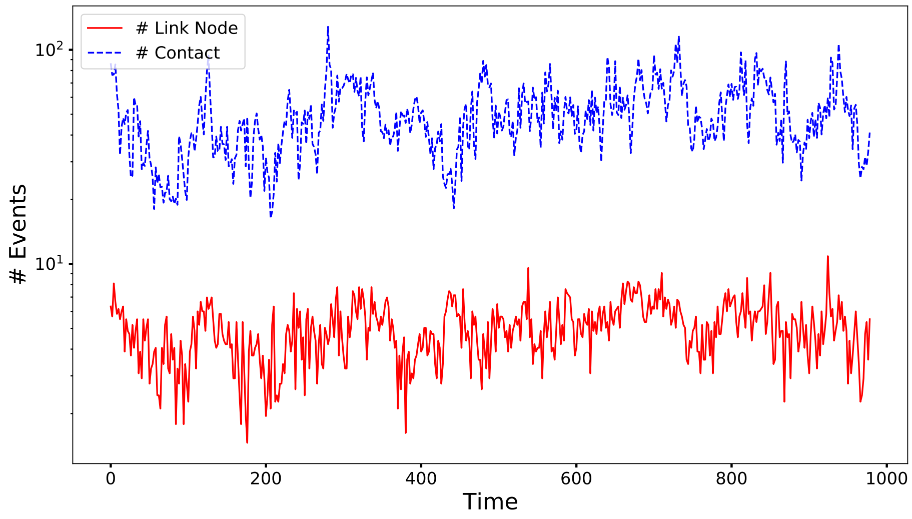

FIGURE S10 The evolution of Link Node and contact events.

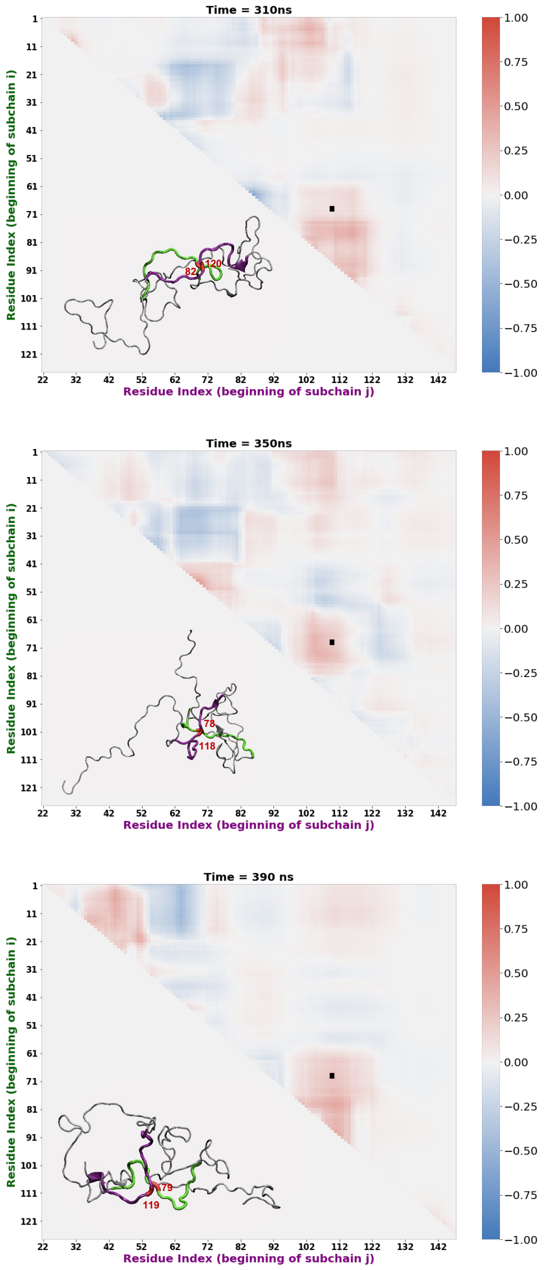

FIGURE S11 A sustained physical link. Dark square: 70-90 and 110-130.

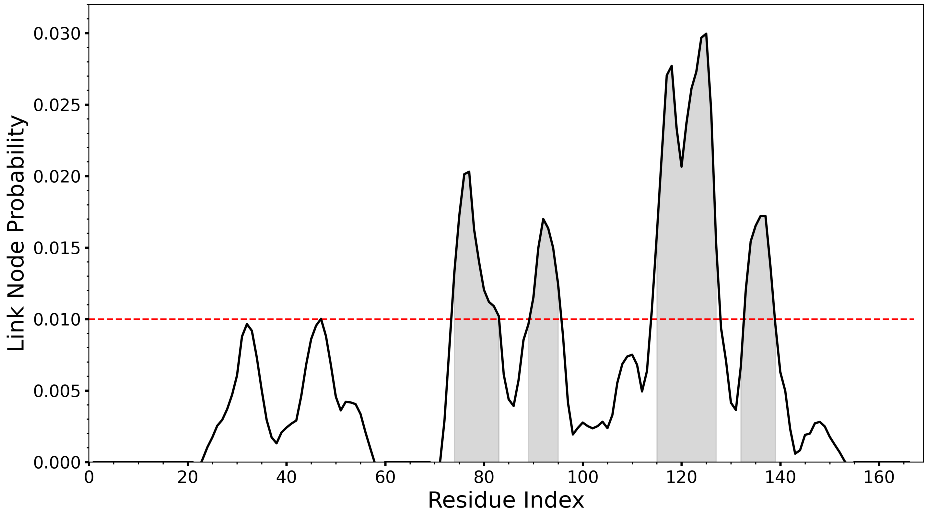

FIGURE S12 The Link Node probability distribution of sustained physical links.

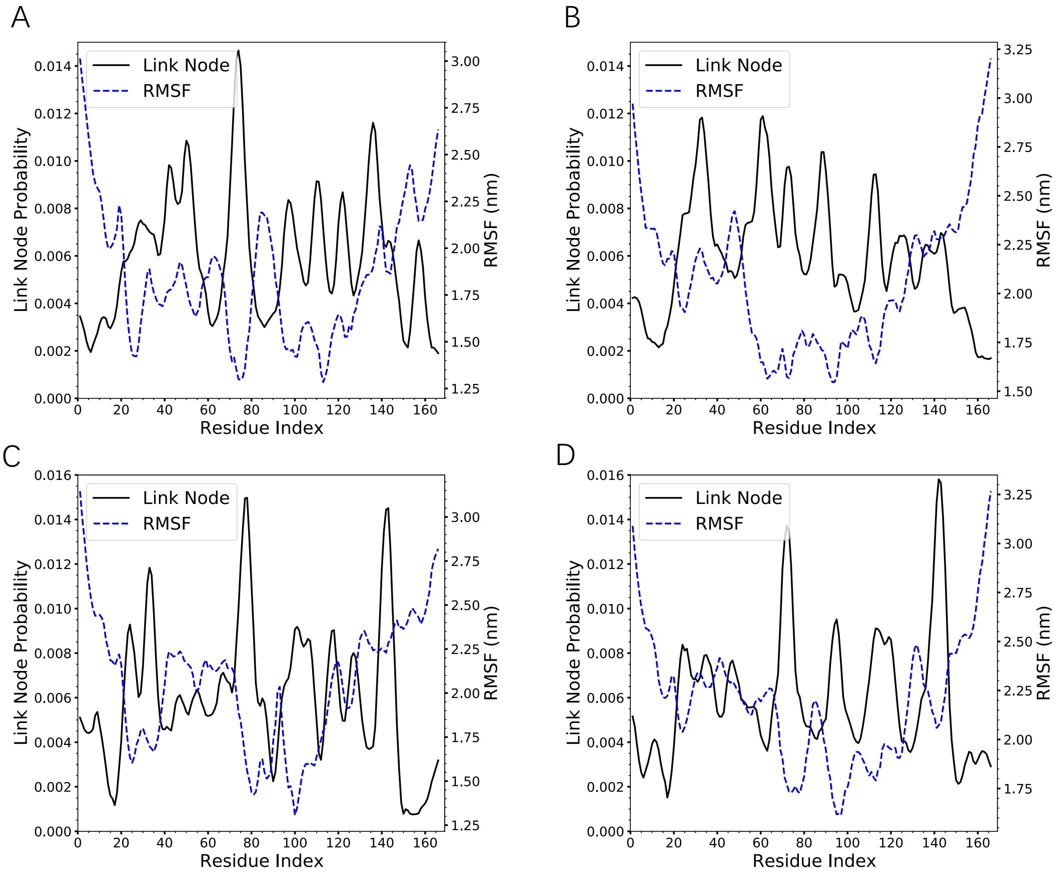

FIGURE S13 The Link Node probability of sequence profiles in other trajectories.

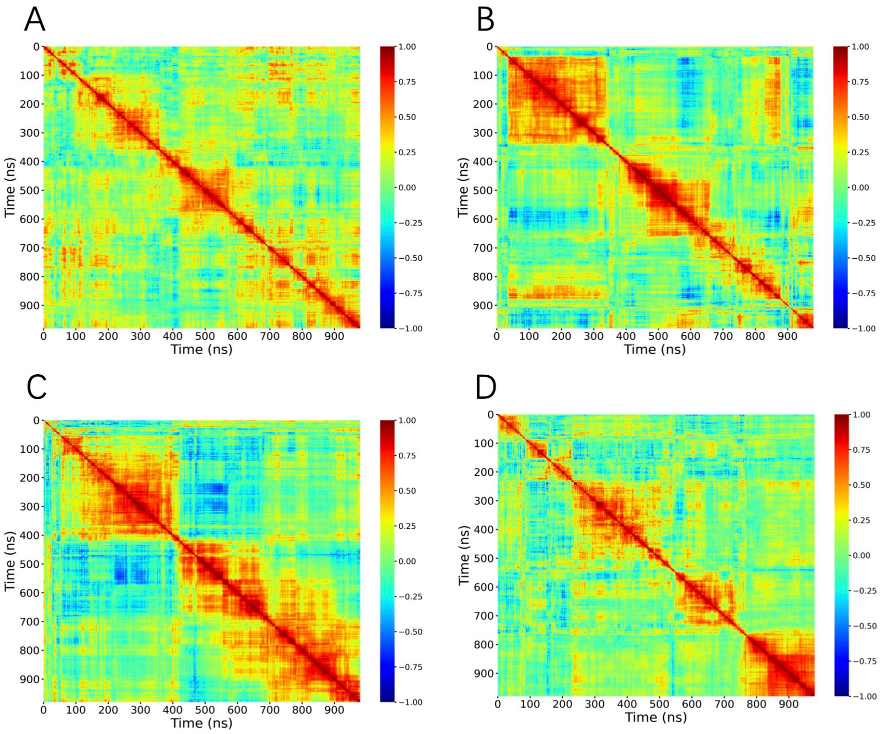

FIGURE S14 The correlation coefficient matrix of GLN Maps of conformations in other trajectories.

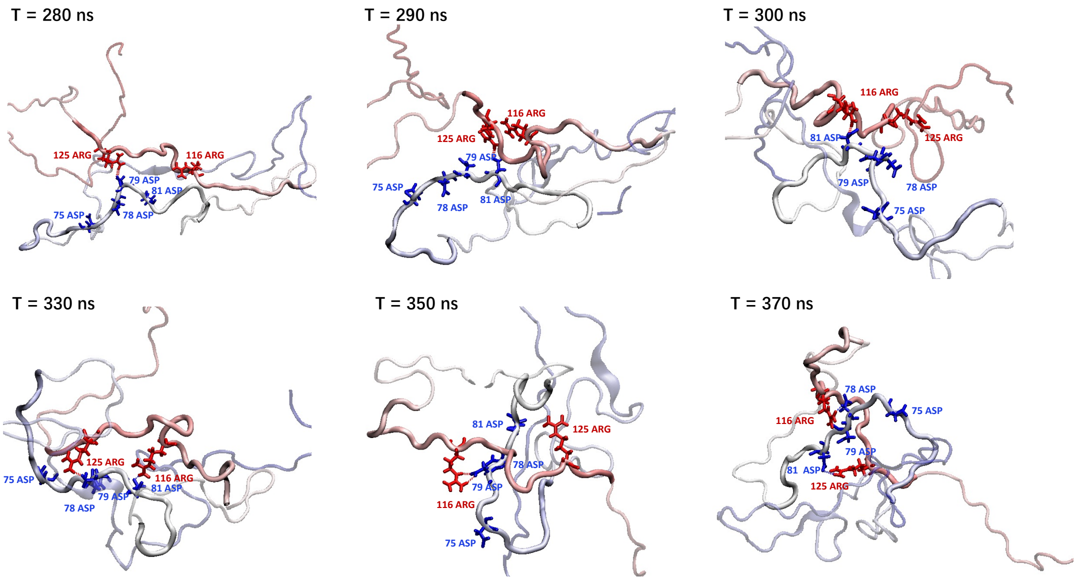

FIGURE S15 The interaction mode between ARG and ASP. The h-bond is in red and the salt bridge is in blue.
